# Outcome Comparability and SWOC (Strength-Weakness-Opportunity-Challenges) Analysis of the National Family Health Survey (NFHS) 1 to 5 for Demographic, Maternal and Child Health, and Behavioral Determinants

**DOI:** 10.7759/cureus.85827

**Published:** 2025-06-12

**Authors:** Lalima Gupta, Raghvendra Gumashta, Rajendra Mahor, Girjesh Gupta, Maharshi N Ojha, Jyotsna Gumashta

**Affiliations:** 1 Community Medicine, People's College of Medical Sciences and Research Centre, Bhopal, IND; 2 Community Medicine, Index Medical College, Hospital and Research Centre, Indore, IND; 3 Physiology, All India Institute of Medical Sciences, Nagpur, Nagpur, IND

**Keywords:** child health, demography, maternal health, nfhs, trend analysis

## Abstract

The effective development of health programs requires the practical application of evidence-based strategies, equitable financial allocation, and the integration of operational research into public health actions. These elements are essential for attaining measurable improvements in healthcare systems and population well-being. By aligning research-driven approaches with policy implementation, we can accelerate progress toward achieving Sustainable Development Goal 3 (SDG-3) targets. This study aimed to engage in an in-depth, evidence-based quantitative and qualitative analysis of National Family Health Survey (NFHS) datasets, as well as a category and sub-category-wise comparability assessment of health indicators. Each round of the NFHS outcomes was analyzed for its application towards programmatic development, implementation, and evaluation. Studies analyzed were representative of each of the studied categories, i.e., demography, maternal health, child health, and behavioral pattern. Literature published under the subheads demography (159), maternal health (286), child health (318), and behavioral pattern (94) and indexed on PubMed Central were analyzed for inferential patterned study. This was followed by the outcome comparability and Strength-Weakness-Opportunity-Challenges (SWOC) analysis of trends, emphasis, and applicability. This trend analysis was quantitatively and qualitatively analyzed.

There was a deviation in sub-criterion identification over various rounds, posing challenges to the use of NFHS data with multiple, varied, and rich resources available for healthcare and support. The lowest expected decline per round compared to their previous rounds in stunting, wasting, and unmet need for family planning was -2.5%, -4.3%, and 1%, respectively. In contrast, the lowest increase per round for female sterilization and antenatal care (ANC) was +1.3% and +1%, respectively. Strengths include the quality of data, sub-categorization over NFHS rounds, use of new tools for assessments, and elimination of some parameters. Weakness was illustrated through unsynchronized conduct of rounds, viz., 6, 7, 10, and 5 years' time gap observed in NFHS-1, 2, 3, 4, and 5, respectively. Opportunity of using data for national development initiatives and fulfilling Sustainable Development Goal-3 can be availed to benefit the healthcare-deprived masses. Threats include intrinsic limitations of data collection, processes of outcome measures for addressing upgradation needs, especially of Reproductive, Maternal, Neonatal, Child Health + Adolescents (RMNCH+A), National Programme for Prevention and Control of Non-Communicable Disease (NPNCD), and national nutrition programs. The policy development, programmatic design, intervention implementation, effectiveness assessment, and National Health Program evaluation need stronger, consecutive, and appropriate evidence datasets for public health action. The felt need of public health intervention can be augmented significantly by using NFHS datasets over time while ensuring adaptable, flexible, and resource-friendly mechanisms of action. Triangulating NFHS datasets, programmatic needs, and financial allocations will hence help create user-friendly public health networks.

## Introduction and background

The National Family Health Survey (NFHS) plays an instrumental role in providing critical and comprehensive data, which shapes health-based social policy making in India. Since its inception in 1992-93, NFHS has evolved into a large-scale survey, providing extensive data and covering a broad range of health indicators. After completing five rounds, the survey has adopted advanced methodology to enhance the accuracy and utility of its findings [1]. The survey offers an in-depth insight into population, health, and nutrition indicators, which are crucial for developing evidence-based public health strategies, especially in maternal and child healthcare [2]. The NFHS is a part of the Demographic and Health Surveys (DHS) program, which is operating in numerous countries [3]. In contrast to the DHS program, the NFHS is tailored uniquely to India’s needs, offering insights at the district level and covering a greater range of the population. With a view to assuring inclusivity and accuracy, methods such as computer-assisted personal interviewing (CAPI) and conducting surveys in local languages are employed, aligning with the practices of other prominent global health surveys such as the Multiple Indicator Cluster Survey by UNICEF.

The NFHS goes a step further by drilling down into critical areas in India, such as nutrition, family planning, and maternal and child health, to address the country’s specific health challenges. It incorporates adaptability to new themes of disability, death registration, and other recent iterations. This enables NFHS to be not only at par with global health surveys but also to uniquely address the specific demography and health landscape of the country [1,2]. Initially, all the states except Sikkim were covered when the survey started in 1992-93. The lessons learnt from the previous rounds have helped enhance and refine the rounds ahead, consequently leading to a successful survey. Five rounds (NFHS-1: 1992-93; NFHS-2:1998-99; NFHS-3: 2005-06; NFHS-4: 2015-16 and NFHS-5: 2019-21) have been completed till date. It has delivered comprehensive insights into the sectors of health, nutrition, and population metrics across the country, encompassing all states and union territories (UTs) [3].

For the latest NFHS round, the survey was conducted in two phases: Phase I: from June 1, 2019, to January 30, 2020, covering 17 states and five UTs; Phase II lasted from January 2, 2020, to April 30, 2021, covering the remaining 11 states and three UTs. Of note, 6,36,699 households, involving 7,24,115 women and 1,01,839 men, were included in the data collection, with response rates of 97.5%,96.9%, and 91.6%, respectively. CAPI was used to conduct the survey in 18 regional languages. Four distinct questionnaires focusing on households, women, men, and biomarkers were used. Samples drawn from primary sampling units (PSUs) were 30,459 from 707 districts; 1061 field teams, comprising a field supervisor, three female interviewers, one male interviewer, two health investigators, and a driver, were deployed [3,4].

The current SWOC (Strength-Weakness-Opportunity-Challenges) cum trend analysis study aimed to conduct an in-depth evidence-based quantitative and qualitative analysis of the NFHS datasets, focusing on category- and sub-category-wise comparability assessment of health indicators.

## Review

Methods

We conducted an in-depth, comprehensive review to evaluate the utility of driving programmatic development, facilitating the on-field implementation of strategies, and assessing the effectiveness of health and social programs in the NFHS rounds. The analysis was conducted across a spectrum of categories, which included demography, maternal health, child health, and behavioral patterns. This ensured a comprehensive representation of critical domains. An exhaustive review for establishing a robust dataset of published literature was also carried out. Of note, 159 publications that focused on demography, 286 on maternal health, 318 on child health, and 94 on behavioral patterns, all sourced from PubMed Central, were identified. The inclusion criteria focused on studies utilizing NFHS data for programmatic development, strategy implementation, and effectiveness assessment of health and social programs. Only peer-reviewed publications from PubMed Central covering demography, maternal health, child health, and behavioral patterns were considered. Studies published in English within a relevant time frame were included. Exclusion criteria included research that lacked direct NFHS data usage, had duplicate findings, was incomplete due to limited data access, was based on non-peer-reviewed sources, was outdated, or was published in a language other than English without a reliable translation. 

The findings from the selected studies were examined critically for their comparability across all five survey rounds. This ensured that trends would be accurately identified and interpreted. Furthermore, SWOC analysis was also conducted to evaluate the significance, implications, and practical applicability of the observed trends. The analysis underscores the key areas where the NFHS outcomes played a pivotal role in influencing policy and programmatic actions. The aspects that required enhancement, as well as potential opportunities for additional research and interventions, were also identified. 

A mixed-methods research was undertaken for the trend analysis, which enables a holistic understanding of the data. The initial focus was on the quantitative analysis, in which numerical patterns and trends were analyzed within the information gathered and depicted through tables and graphs generated through MS Excel. This helped in assessing the increasing or decreasing trend. Simultaneously, in the Qualitative analysis, the assessment and interpretation of the contextual factors that might influence the trends, such as cultural practices, socioeconomic conditions, or policy interventions, were performed. 

Results

Demographic indicators have been the cornerstone of the NFHS for all the subsequent surveys. These have acquired more significance in the NFHS-4 and 5. The number of demographic indicators collected in NFHS-4 almost tripled, and in NFHS-5, it almost quadrupled as compared to the earlier surveys. A similar trend of increasing emphasis has been noted for child health and maternal health. Child health emerged as the top priority (32 indicators), followed by maternal health (27 indicators) and demography (19 indicators) during the survey year 2019-21 (NFHS-5). The highest focus on maternal health (14 indicators) occurred between 1998 and 99 (NFHS-2). The emphasis on maternal health and child health remained equal during NFHS-3 (15 indicators) and NFHS-4 (27 indicators). Over time, the surveys have changed their focus as per the need of the hour. Initial attention was paid to demographics, which would have been a key driver to understand the population dynamics. The focus then shifted to maternal health. By the most recent survey in 2019-21, i.e., the NFHS-5, larger emphasis was placed on child health, with the highest number of indicators collected in the area. The trends related to the emphasis placed on demographics and maternal and child health are presented in Table 1.

**Table 1:** Trends of emphasis on demography and maternal and child health indicators during NFHS rounds The table includes the number of indicators for demography and maternal and child health in the NFHS. The increasing trend in the indicators can be seen from NFHS-1 to NFHS-5, which reflects its adaptive nature to the recent needs NFHS: National Family Health Survey

	NFHS-1	NFHS-2	NFHS-3	NFHS-4	NFHS-5
Demography	5	8	8	17	19
Maternal health	7	14	15	27	27
Child health	9	11	15	27	32

**Figure 1:**
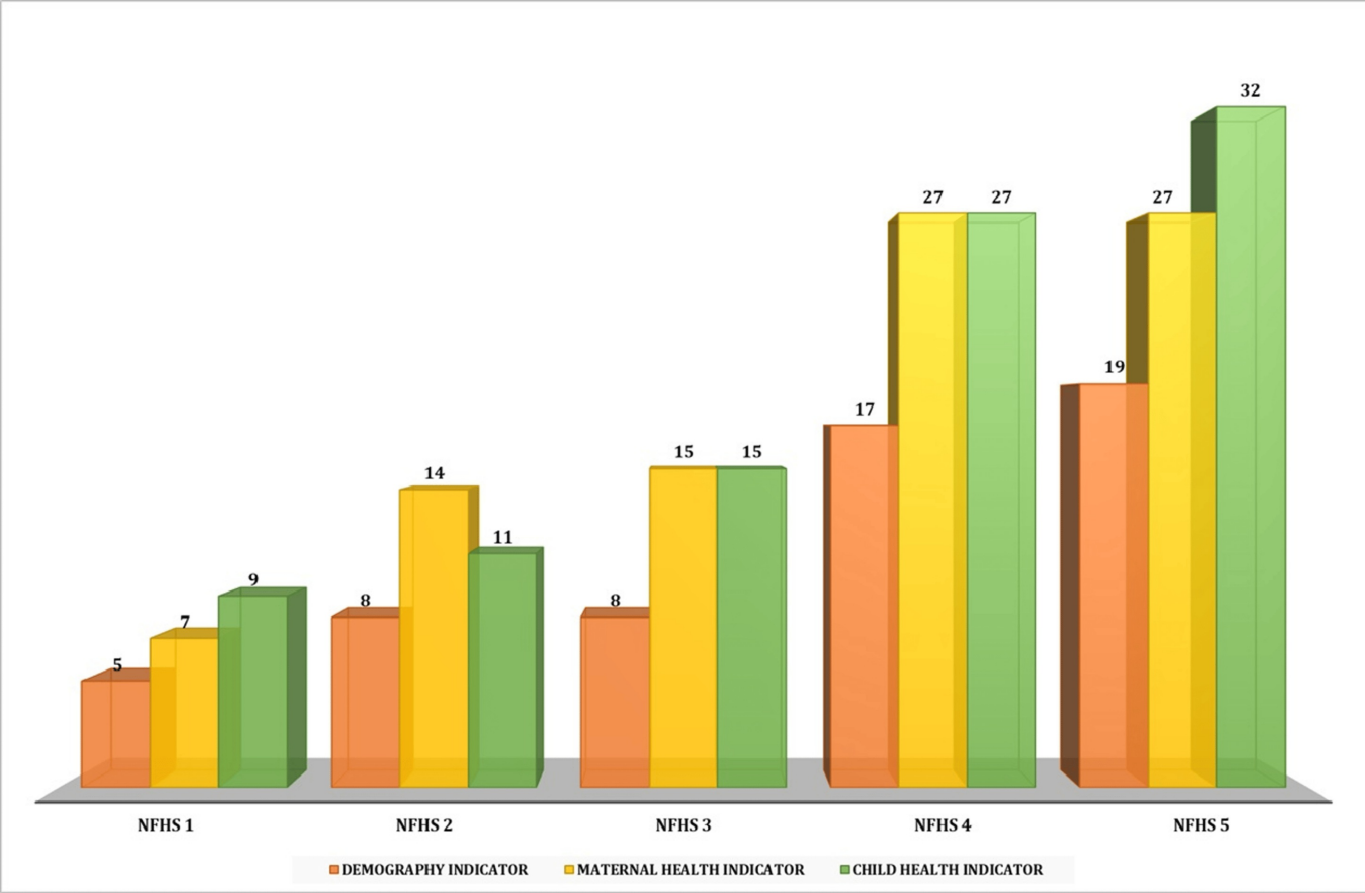
Number of indicators for demography and maternal and child health in NFHS rounds NFHS: National Family Health Survey

Figure 1 provides a graphical representation of the number of indicators for demography and maternal and child health in various NFHS rounds for comparative analysis.

Table 2 summarizes the baseline and subsequent status of various parameters in NFHS rounds.

**Table 2:** Baseline and subsequent status of various parameters in NFHS rounds NFHS: National Family Health Survey

	NFHS-1 (%)	NFHS-2 (%)	NFHS-3 (%)	NFHS-4 (%)	NFHS-5 (%)
Unmet needs of family planning	19.5	15.8	13.9	12.9	9.4
Antenatal care	62.3	65.4	37	51.2	58.1
Underweight prevalence	53.4	47	42.5	35.8	32.1
Stunting	52	45.5	48	38.4	35.5
Wasting	17.5	15.5	19.8	21	19.3
Female sterilization	30.8	36	37.3	36	37.9

Table 3 presents the round-wise variations in key indicators in NFHS. 

**Table 3:** Round-wise variations in key indicators in NFHS The table presents the round-wise variations in key indicators of NFHS. It compares select criteria—including the unmet need for family planning, antenatal care, underweight prevalence, stunting, wasting, and female sterilization—across different survey rounds. Indicators that ideally should have improved over time but showed a decline are marked with a negative sign, while those demonstrating positive progress are denoted otherwise NFHS: National Family Health Survey

	NFHS-2 (%)	NFHS-3 (%)	NFHS-4 (%)	NFHS-5 (%)
Unmet needs of family planning	+3.7	+1.9	+1	+3.5
Antenatal care	+3.1	-27.4	+14.2	+6.9
Underweight prevalence	+6.4	+5.5	+6.7	+3.7
Stunting	+6.5	-2.5	+9.6	+2.9
Wasting	+2	-4.3	-1.2	+1.7
Female sterilization	+5.2	+1.3	-1.3	+1.9

There was a deviation in sub-criteria identification over various rounds. The difference in consistency poses challenges in effectively utilizing the NFHS data alongside multiple, diverse, and rich resources available for health care and support. The lowest expected decline per round as compared to their previous rounds in stunting, wasting, and unmet need for family planning was -2.5%, -4.3%, and 1%, respectively. In contrast, the lowest increase per round for female sterilization and ANC care was 1.3% and +1%, respectively.

Strength-Weakness-Opportunity-Challenges (SWOC) Analysis of the National Family Health Survey

The NFHS offers a comprehensive perspective on India's health and demographic growth. Its SWOC analysis highlights the strengths and limitations of this monumental effort over its five rounds. NFHS is known as one of the largest surveys globally in terms of respondents. It provides significant information at the national, state, and even district levels. By the fourth and fifth rounds, the survey expanded to cover district-level data for 707 districts, offering policymakers complex insights for targeted interventions. The NFHS collects data on key areas like maternal and child health, nutrition, population dynamics, family planning, and household conditions. This detailed information has been beneficial in designing programs such as the Janani Suraksha Yojana and the National Nutrition Mission, ensuring thorough guidelines in all aspects.

Every round of NFHS has incorporated technical advancements. From paper-based surveys in the early rounds to the adoption of CAPI in NFHS-4 and NFHS-5, the trends in techniques have enhanced data quality and specificities. Interviews were conducted in 18 different languages (regional and local) to ensure that respondents across India’s varied semantic spectrum can participate adequately. Insights from NFHS data have helped shape key national programs like the National Health Mission (NHM) and Beti Bachao Beti Padhao. The focus on maternal and child health has informed vaccination drives, nutrition interventions, and safe delivery practices. The survey also influences international collaborations and comparisons, contributing to global health goals. As part of the DHS program, NFHS aligns with international data standards, facilitating comparability and credibility. This recognition has established NFHS as a reliable repository of demographic and health insights.

The NFHS's expansive scope poses formidable logistical challenges, particularly in remote and conflict-ridden areas, where access and respondent cooperation can be weak. Data accuracy is frequently compromised by recall bias, cultural stigma, and underreporting, which can undermine the survey's validity. Delays in processing and disseminating findings can attenuate their immediate policy impact, as evidenced by NFHS-4 (2015-16) and NFHS-5 (2019-21). Although the survey has broadened its scope over time, critical issues like mental health, non-communicable diseases (NCDs), and environmental factors were incorporated only in later rounds, highlighting the need for ongoing refinement.

The NFHS's massive scale necessitates substantial resources, raising concerns about long-term sustainability. Harnessing digital tools, artificial intelligence, and mobile surveys could expedite data collection and analysis, enhancing the survey's efficiency. Expanding the survey's focus to cover modern health concerns like climate change and digital healthcare would augment its relevance and applicability. Training local data collectors, particularly in rural and tribal areas, can improve data quality and its application in governance, fostering more informed decision-making.

Encouraging state governments, NGOs, and private organizations to leverage NFHS findings can drive localized health initiatives, promoting more targeted and effective interventions. Collaborations with academic institutions can further research, while adopting global best practices can refine NFHS methodologies and establish it as a paradigm for large-scale health surveys worldwide.

Discussion

The policy implications and development, intervention implementation, programmatic design effectiveness assessment, and National Health Program evaluation require robust, evident data sets for evidence-based public health action. NFHS, through the five successful rounds, has given significant and invaluable insights into India's transitioning demographic health dynamics and landscape, pinpointing the demanding areas of concern. Examining the trends across successive surveys reveals a meaningful shift in focus toward maternal and child health [4].

The sub-categorization of NFHS rounds, the use of new tools of assessment, the quality of data, and the elimination of some parameters were identified as the strengths. Weakness was illustrated through asymmetrical and unsynchronized conduct of the NFHS rounds, viz., 6, 7, 10, 5 years’ time gap was observed in NFHS-1, 2, 3, 4, and 5, respectively. The leading opportunity of using robust data for national development initiatives, aligning with Sustainable Development Goal 3 (SDG-3) targets, can be redeemed to the benefit of the destitute or the health-deprived masses. The threats included the intrinsic limitations of data collected, and processing of the outcome measures for addressing upgradation needs, especially of the national nutritional programs, RMNCH+A, and NPNCD [4,5,6,7].

In the latest NFHS-5, child health stood out with the highest attention, represented by 32 indicators, which was followed by maternal health and demography indicators, having 27 and 19 indicators each, respectively. The prioritization in these fields reflects an in-depth and concurrent understanding of the essential role that maternal and child well-being play, along with the lasting impact these areas have on society. The earlier surveys leaned heavily on demographic aspects. The latest rounds emphasized maternal and child health, also addressing nutrition, reflecting a step closer to breaking the vicious cycle of malnutrition, which mirrors a thoughtful responsiveness to the nation’s evolving health needs. It’s a testament to how the NFHS survey adapts to prioritize the well-being of the most vulnerable, which ensures a brighter and healthier future for all.

The SWOC analysis has further provided the much-needed clarity. The NFHS's strengths included the high-quality, granular data collection across the spectrum of the diverse domains, which were supported by methodical technological advancements like CAPI and its harmonization with global standards under the DHS program. The findings from NFHS have enlightened the current initiatives like the National Nutrition Mission, including the POSHAN Abhiyaan and the Beti Bachao Beti Padhao, along with other social schemes, underlining the policy impact. Nevertheless, the weaknesses stated as irregular intervals between rounds and variability in data collection parameters underscore the need for greater synchronization and standardization [5]. The significant opportunities lie in the integration of advanced data analytics, addressing emerging health challenges such as climate change and the upcoming pandemic of NCDs, and fostering localized data utilization. However, challenges lie in the resource constraints and inherent limitations in data collection processes, necessitating deliberate attention.

The contributions of the NFHS rounds have faced several challenges, which include variability in types of and number of indicators used, posing difficulty in longitudinal and standardized comparisons. The observed deviation in sub-criteria identification underscores this complexity. In comparison with the previous round, the survey has recorded incremental progress, such as declines in stunting, wasting, and unmet family planning needs, as well as modest increases in female sterilization and antenatal care coverage. These improvements underscore the need for sustained efforts to close persistent health gaps [4].

The rising burden of severe acute malnutrition (SAM) across NFHS rounds provides insights into the urgency of strengthening Anganwadi centers, expanding social, health, and educational networks for nutritional support, and maintaining food security mechanisms and quick referral services for rehabilitation of the malnourished. This also calls for the need for extensive nutritional counseling to the mother during the antenatal care and post-natal care periods [6,8,9]. Such actions are critical in achieving sustained improvements in under-5 health outcomes. Additionally, significant inter-pregnancy intervals have been linked to better child health outcomes, as they allow for adequate caregiving and recovery between pregnancies. Policies promoting extended inter-pregnancy intervals can thus play a vital role in reducing under-5 morbidity and mortality [8,10].

The survey data also indicates that though poverty remains a major contributor to the disease burden, other factors like education, housing, diet, nutrition and sanitation, and an enabling environment have emerged as key factors in driving better health outcomes. These determinants accentuate the wider social and cultural context underpinning health advancements, calling for holistic and inclusive interventions. Furthermore, the observed decline in mean height among women aged 15-25 years, particularly among disadvantaged and tribal communities, reflects the combined effects of urbanization, malnutrition, and lifestyle changes. Addressing these disparities and necessitates requires targeted interventions tailored to marginalized populations [7].

Issues such as non-sampling errors and outlier biases in data collection, particularly concerning newborns and under-five children, have also been identified [8,11]. Improvement in these challenges is essential for ensuring the reliability and consistency of NFHS data and guiding effective public health strategies. It is equally crucial to develop inferential parameters for equitable representation, accounting for diverse socio-demographic indicators and population densities, to produce balanced estimates and enhance the survey's overall utility [12]. Distresses regarding the accuracy and comparability of child growth monitoring charts used in the NFHS survey underscore the need to reconsider and refine these tools to reflect the unique characteristics of India's population [8,11,13,14]. The strengthening of growth monitoring systems will further bolster efforts to tackle malnutrition and improve child health outcomes, and ultimately improve the overall health status of the country.

The limitations, such as inconsistent time gap between rounds, variability in indicators, non-sampling errors, outlier bias, data processing, and outcome, etc., underscore the need for improved standardization, refined data collection method and enhanced resource allocation to ensure that the NFHS remain a robust tool for evidence based public health strategies. 

Road Ahead: Future Opportunities

The unmet need for family planning, especially in hard-to-reach areas, can be managed through a holistic approach by including not only the spouse in counseling for the family planning but also the other near family members who act as passive influencers to the need for family planning [15].

The undernutrition aspect can be further addressed in different geographical regions by mapping the underprivileged areas and tracking the nutritional status completely from birth till early adulthood. This would not only be effective in supervision and needs assessment but also in breaking the vicious cycle of malnutrition [16]. A supplement to the Mother-Child Tracking system can be established for such monitoring. The result from such policies will serve as a catalyst for an evidence-based framework and policies for further programmatic management in the under-performing geographical region [17]. Achieving measurable progress in the undernutrition indicator, which would also reflect in the SDGs, across various initiatives, will require collaborative actions from both the state and national levels. It is also imperative to closely examine the principal findings of the National Family Health Survey and juxtapose them with the results from prior surveys to discern discernible trends, whether positive or negative, in reported data indicators [18]. 

Finally, robust monitoring and evaluation mechanisms are essential to track progress, identify gaps, and ensure accountability, ultimately paving the way for a healthier and more nourished India [19,20]. The amalgamation of the forthcoming rounds of the NFHS with artificial intelligence in health technology can lead to enhanced, cleaner, and reliable data. This will further lead to a simultaneous tailored intervention rather than a one-size-fits-all approach and strategies to overcome the gaps in the public health system. It will also assist in analyzing the vast data sets efficiently and also can cover can uncover subtle predictions and health outcomes. The issue of data privacy and accuracy, if addressed, will pave the way for a more proactive, precise, and responsive public health system, ultimately leading to significant improvement in health outcomes across India and better health for all.

## Conclusions

The NFHS has played a key role in transforming India’s health and demographic landscape through in-depth insights and robust data in five rounds. This analysis highlights the urgent need to establish a fixed, predictable schedule for the NFHS, perhaps through legislative action, to ensure that data can be used for consistent and timely monitoring of national health goals. The concurrent evolution of the survey points to its ability to adapt to the country’s dynamic health priorities, shifting focus from the demographic indicators to major emphasis on maternal and child health. Triangulation of NFHS data along with artificial intelligence, financial allocations, and programmatic objectives highlights an opportunity to build a more integrated and user-centric, and friendly public health network. This approach ensures that interventions are tailored to the specific requirements of communities, maximizing their impact. The NFHS findings can also be used to identify priority areas, enabling targeted efforts to address critical health challenges.

Furthermore, integrating NFHS data with a real-time programmatic feedback system will allow for iterative improvements in policies and strategies, ensuring that resources are allocated effectively and interventions remain adaptable to changing health scenarios, including emerging concerns like climate change and the rise of NCDs. A dynamic framework like such that fosters the development of innovative solutions that are both sustainable and scalable, ensuring long-term benefits for public health. Ultimately, the NFHS continues to be a cornerstone of health policy and planning in India, providing a robust framework for crafting inclusive, impactful, and sustainable interventions that contribute to a healthier future for all.
